# Association of Moderate Beer Consumption with the Gut Microbiota and SCFA of Healthy Adults

**DOI:** 10.3390/molecules25204772

**Published:** 2020-10-17

**Authors:** Natalia González-Zancada, Noemí Redondo-Useros, Ligia E. Díaz, Sonia Gómez-Martínez, Ascensión Marcos, Esther Nova

**Affiliations:** Immunonutrition Group, Department of Metabolism and Nutrition, Institute of Food Science, Technology and Nutrition (ICTAN), Spanish National Research Council (CSIC), C/Jose Antonio Novais, 28040 Madrid, Spain; nataliagonzalez_91@hotmail.com (N.G.-Z.); noemi_redondo88@hotmail.com (N.R.-U.); ldiaz@ictan.csic.es (L.E.D.); sgomez@ictan.csic.es (S.G.-M.); amarcos@ictan.csic.es (A.M.)

**Keywords:** alcohol, butyric acid, fiber, polyphenols, drinking pattern

## Abstract

Fermented alcoholic drinks’ contribution to the gut microbiota composition is mostly unknown. However, intestinal microorganisms can use compounds present in beer. This work explored the associations between moderate consumption of beer, microbiota composition, and short chain fatty acid (SCFA) profile. Seventy eight subjects were selected from a 261 healthy adult cohort on the basis of their alcohol consumption pattern. Two groups were compared: (1) abstainers or occasional consumption (ABS) (*n* = 44; <1.5 alcohol g/day), and (2) beer consumption ≥70% of total alcohol (BEER) (*n* = 34; 200 to 600 mL 5% vol. beer/day; <15 mL 13% vol. wine/day; <15 mL 40% vol. spirits/day). Gut microbiota composition (16S rRNA gene sequencing) and SCFA concentration were analyzed in fecal samples. No differences were found in α and β diversity between groups. The relative abundance of gut bacteria showed that *Clostridiaceae* was lower (*p* = 0.009), while *Blautia* and *Pseudobutyrivibrio* were higher (*p* = 0.044 and *p* = 0.037, respectively) in BEER versus ABS. In addition, *Alkaliphilus,* in men, showed lower abundance in BEER than in ABS (*p* = 0.025). Butyric acid was higher in BEER than in ABS (*p* = 0.032), and correlated with *Pseudobutyrivibrio* abundance. In conclusion, the changes observed in a few taxa, and the higher butyric acid concentration in consumers versus non-consumers of beer, suggest a potentially beneficial effect of moderate beer consumption on intestinal health.

## 1. Introduction

Alcoholic beverage consumption, and its effects on health, is nowadays a controversial topic, with no clear-cut and widely accepted recommendations readily available for all circumstances, even on a population group basis. While scientific evidence on regular and moderate consumption of wine and beer have shown benefits for the risk of cardiovascular disease [1], health organizations claim that the possible benefits do not outweigh the risks, and avoiding alcohol is the best choice for those who are not habitual consumers. Thus, investigations on the impact of these beverages on health are certainly needed. Beer is the most widely consumed alcoholic beverage throughout the world, and contains a multitude of different compounds, many of which are produced during the fermentation process. Some minerals and vitamins such as fluoride, silicon, choline, and folate are present in significant amounts, so that two cans might provide 10% of the recommended dietary allowance (RDA) [1]. Polyphenols from malt and hop are important active compounds in beer that confer antioxidant activity, and can act synergistically with dietary constituents. Beer is also a source of dietary fiber, mainly composed of β-glucans from barley and arabinoxylo-olygosaccharides (AXOS) [2]. These compounds or their combination, i.e., hydrolysable polyphenols which are non-extractable with aqueous organic solvents [3], are mainly conducted undigested to the lower gut and are metabolized by gut bacteria. The symbiotic relationship between the gastrointestinal tract and the gut microbiota has a key role for human health, and many factors can influence this relationship such as lifestyle, environmental factors, the ageing process, etc. While the intestine provides nutrients and good conditions for the gut microorganisms to thrive [4], these microorganisms participate in energy extraction from food and the synthesis of vitamins and aminoacids, in addition to building defensive barriers against pathogens [5,6]. Furthermore, disruption of the normally stable microbial communities, known as gut dysbiosis, is accompanied by variable inflammatory conditions.

Diet is a particularly important factor for intestinal homeostasis and health, and affects greatly the composition and abundance of the microbial community. Regarding alcoholic drinks, separating the effects of ethanol from those of the raw plant components (e.g., polyphenols and fiber) and those formed in the technological and maturation processes of beer making seems a useful research strategy. Evidence in the literature demonstrates that alcohol consumption can lead to quantitative and qualitative dysbiosis in the intestine of rodents and humans [6]. In general, chronic alcohol consumption is associated with bacterial overgrowth, a decrease of *Bacteroidetes,* and an increase of *Proteobacteria* and *Fusobacteria*, endotoxin translocation, and inflammation, especially in alcoholic liver disease patients [6]. However, alcoholic beverages consumed in moderation should be examined as a complex intake, since the ethanol delivery would be within the low range, and its detrimental effects, if existent at all, might be overruled by the beneficial effects of the bioactive compounds present, for example, in fermented drinks, such as wine or beer. In this sense, the fermentation process in the gut yields energy for microbiotic proliferation and metabolite production, e.g., short chain fatty acids (SCFA) [7] for regulation of inflammatory responses [8] and gut hormone secretion [9] in the host [5]. Regarding gut microbiota composition, the dietary administration of red wine polyphenols in a mouse model of carcinogenesis changed the fecal microbiota composition to a predominance of *Bacteroides, Lactobacillus,* and *Bifidobacterium* spp., compared to the most abundant *Bacteroides, Clostridium,* and *Propionibacterium* spp. in the control animals [10]. Despite this finding, the number of published studies on fermented alcoholic beverages and gut microbiota is scarce, both in animals and humans, especially considering moderate alcohol consumption. However, some studies have proven the interactions between beer polyphenols and gut microbiota, and have been recently summarized in a review [11]. Thus, results on the most widely studied flavonoids, such as quercetin or catechin, which are present in beer, have shown their capacity to modulate gut microbiota. Tzounis et al. showed in an in vitro model that (−)-epicatechin and (+)-catechin promote the growth of the *Clostridium coccoides-Eubacterium rectale* group, and inhibit the growth of the *Clostridium histolyticum* group [12]. In addition, isoxanthohumol present in hops, together with other prenylated flavonoids, can be metabolized to render 8-prenylnaringenin, through an O-demethylation that is carried out by *Eubacterium limosum* [13]. Moreover, metabolization by *Eubacterium ramulus* transforms prenylated flavonoids into its chalcones, and likely affects both the activity and toxicity of ingested molecules [14].

Regarding dietary fiber in beer, an interesting study fed rats with different diets containing fiber from barley malts, brewer’s spent grain, and barley extracts, resulting in varying amounts of β-glucan, soluble arabinoxylan, and insoluble arabinoxylan in the diets [15]. The results showed that there is a potential to stimulate butyrate- and propionate-producing bacteria in the cecum of rats with malt products of specific fiber properties, compared with a fiber-free diet. The authors also pointed out that a complex mixture of fiber, as in the malts, is of greater importance for microbiota diversity than purer fiber extracts. A different study in high-fat fed rats showed that barley and barley malt differed in the microbiota modulatory effects, compared to values in the cecum of control rats. Those fed malt showed higher *Roseburia, Coprococcus,* and *Lactobacillus*, which was related to the changing characteristics of β-glucans during malting, while barley was associated with higher *Blautia* and *Akkermansia* [16].

SCFAs are formed during the fermentation of undigested carbohydrates by the lower gut microbiota [17]. The main SCFAs produced (acetate, propionate, and butyrate) are rapidly absorbed by the colonocytes, and used as an energy source and precursors in anti-inflammatory mechanisms [17]. In addition, SCFAs are key substrates in the cross-feeding web, which comprises the intestinal microbiota. As an example, mutual cross-feeding interactions occur between *Bifidobacterium longum* and *Eubacterium rectale*. Both strains consume AXOS, but the bifidobacterial strain is additionally stimulated by consuming the monosaccharides released by the extracellular degradation of AXOS by the *E. rectale* strain, leading to cross-feeding interactions that are mutually beneficial [18]. Moreover, *Faecalibacterium prausnitzii* uses lactate produced by certain *Bifidobacterium* spp. to produce butyrate [19]. In the presence of AXOS, Bifidobacteria and butyrate-producing colon bacteria (*F. prausnitzii, Eubacterium*, and *Roseburia* spp.) are stimulated simultaneously, with a significant increase of butyrate production as a result [18].

Despite the above evidence that beer components could potentially have an influence on gut microbiota composition, there is a gap in research regarding beer consumption and microbiota modulation in humans. Only one intervention study has been published so far aimed at studying the effects of both, non-alcoholic and alcoholic beer on the microbiota of healthy adults, which were 89% between 21 and 35 years old [20]. The results showed that non-alcoholic beer increased the diversity of the microbiota after 30 days of intervention, while alcoholic beer did not, but both favored the proliferation of the *Bacteroidetes* phylum in relation to the *Firmicutes* phylum. Significant changes were observed in the relative abundance of a number of taxa with both types of beer, which in the case of non-alcoholic beer the authors propose as enrichment with beneficial bacteria [20]. Thus, in order to reduce the shortage of information published in this field, the aim of this work was to study the associations between beer consumption and gut microbiota composition in healthy adults as well as the concentration of SCFA. To this end, men and women selected from a larger cohort were studied in two groups, differing in their alcohol consumption pattern. The first group included abstainers, and the second group subjects consuming beer as their fundamental alcoholic drink choice.

## 2. Results

### 2.1. Anthropometric, Lifestyle, and Dietary Profile Characteristics of the Beer Consumption Groups

As observed in Table 1, the men in the abstainers (ABS) group showed a higher body mass index (BMI) than that of the subjects in the beer consumption (BEER) group (*p* = 0.038). Furthermore, they also showed a tendency to have higher body fat and visceral fat (both *p* = 0.057). On the other hand, the women in the BEER group tended to show higher total dietary energy consumption (kcal/d) than women in the ABS group (*p* = 0.054; Table S1). Although most of these results are trends, these variables were taken into account as indicated in the statistical methodology when analyzing the significant differences in the bacterial taxa studied. Regarding alcohol consumption by type of drink, the median of beer consumption, in men of the BEER group was 11.6 alcohol g/day and in women was 13.5 alcohol g/day, which is 232 and 270 mL of beer in men and women, respectively. In addition, the median for alcohol consumption from wine and spirits in men was 0 and 1 alcohol g/day, equivalent to 2 mL spirits per day. In women of the BEER group, these were 0.39 and 0.36 alcohol g/day respectively, equivalent to 3 mL and 0.9 mL of wine and spirits per day, respectively (Table S2).

### 2.2. Gut Microbiota Diversity and Relative Abundance

**Figure 1:**
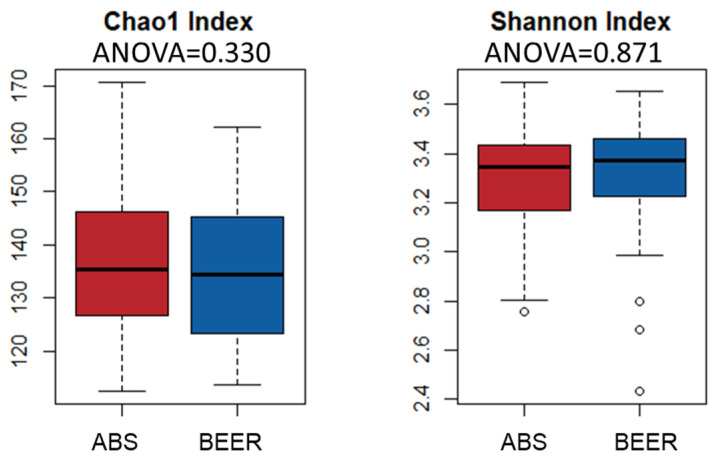
Differences in α diversity indices (Chao1 and Shannon) between beer consumption groups. ANOVA test.

The analysis of α diversity showed that the Chao1 index (richness) and the Shannon index were similar between subjects consuming beer and abstainers (*p* = 0.330 and *p* = 0.871, respectively) (Figure 1). No differences were found, either, in β diversity (*p* = 0.332) (Figure S1) between beer consumption groups in a principal coordinates analysis (PCoA), based in the Bray-Curtis distance matrix with permutational multivariate analysis of variance (PERMANOVA) test.

The relative abundances of phyla, families, genera, and species that can metabolize beer components were selected from the metagenomic sequence analysis aggregate tables and compared between alcohol consumption groups. No differences in phyla abundances were observed between the groups (Table S3). Moreover, out of the eight bacterial families studied, differences were only observed in the *Clostridiaceae* family (*p* = 0.009), which presented a lower relative abundance in the BEER group compared to the ABS group (Table 2, Figure S2).

**Figure 2:**
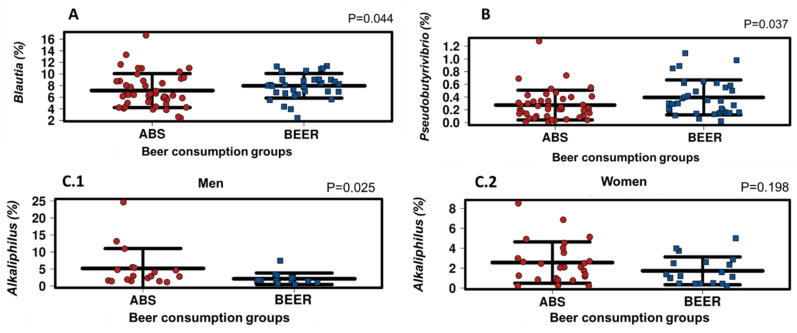
Differences between beer consumption groups in the abundance of the genus Blautia (**A**), Pseudobutyrivibrio (**B**), and Alkaliphilus (**C**). The Alkaliphilus genus is stratified in men (**C.1**) and women (**C.2**). MW-U test. Significance at *p* < 0.05.

Regarding genera (Table 3), *Blautia* and *Pseudobutyrivibrio* showed higher levels in the BEER group compared to the ABS group (*p* = 0.044 and *p* = 0.037, respectively) (Figure 2A,B). Furthermore, *Alkaliphilus* showed lower levels in the BEER group compared to the ABS group, but only in men (*p* = 0.025) (Table 3; Figure 2C.1). Finally, non-significant trends were found for lower levels of *Clostridium* genus (*p* = 0.056), and higher levels of *Johnsonella* (*p* = 0.051) and *Butyrivibrio* (*p* = 0.056) in the BEER group compared to the ABS group.

Regarding species analysis within the genera that differed between consumption groups, the results showed that *Alkaliphilus peptidifermentans* (*p* = 0.028) and *Clostridium hiranonis* (*p* = 0.006) were less abundant in the BEER group than in the ABS group (Table 4; Figure S3A,B, respectively). In contrast, *Blautia coccoides* (*p* = 0.027), *Pseudobutyrivibrio xylanivorans* (*p* = 0.037), *Johnsonella ignava* (*p* = 0.046), and *Blautia producta* (*p* = 0.039) showed higher levels in the BEER group compared to the ABS group (Table 4; Figure S3C–F, respectively).

### 2.3. SCFA Concentration in the Gut

Since SCFAs could be produced following the fermentation of beer components by the microbial taxa analyzed, their concentration was measured in feces. A higher concentration of butyric acid was observed in the BEER group compared to the ABS group (*p* = 0.032; Figure S4), while the rest of the SCFA analyzed did not show significant differences (Table 5).

**Figure 3:**
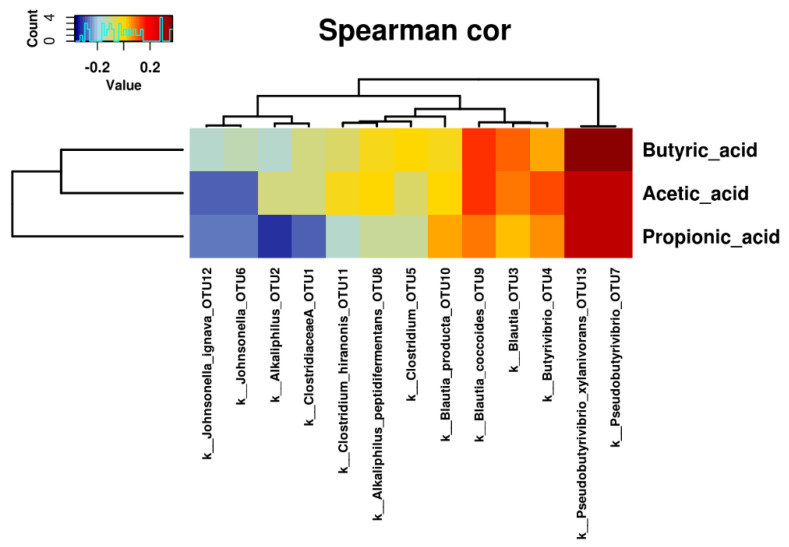
Heatmap representation of correlation coefficients between SCFA concentrations and gut microbiota composition. Only taxa with significantly different abundance in both beer consumption groups were included.

The correlations between the abundance of those taxa showing different levels in beer consumers and abstainers and the SCFA concentrations were analyzed. As observed in Figure 3, the three main SCFAs, acetic, propionic, and butyric acids, were positively correlated to *Pseudobutyrivibrio* and *Pseudobutyrivibrio xylanivorans* (all *p* < 0.015). On the other hand, acetic and propionic acids were negatively correlated, although with marginal significance, to *Johnsonella* and *Johnsonella ignava* (all *p* < 0.040). Furthermore, propionic acid was also negatively correlated with *Clostridiaceae* and *Alkaliphilus* (both *p* < 0.010).

## 3. Discussion

Beer is the most abundantly consumed drink among alcoholic beverages, but its effects on health are still controversial. Given the high proportion of alcohol abuse in many populations and the high prevalence of consumption patterns that fall far from the “moderate and regular”, stronger evidence on the healthy properties of beer or its components is warranted, in order to balance recommendations for this ancient drink. This work analyzed the changes in the intestinal microbial groups that could be associated with regular and moderate consumption of beer as compared to abstinence. The study was directed towards those taxa that could use some of the components of beer as a metabolic substrate, such as fiber and polyphenols. A few taxa showed changes between consumption groups, which together with the higher concentration of butyric acid in consumers versus non-consumers of beer, suggest that the effect of beer consumption might be beneficial for intestinal health, and deserves further investigation.

When studying possible demographic, anthropometric, or lifestyle differences of the volunteers, a high similarity was found in most of the variables between the BEER and ABS groups. It should be noted, as an exception, that abstemious men showed a trend towards a higher percentage of body fat, specifically visceral fat. For this reason, the composite variable “BMI-fat” was considered as a potential confusion factor in the subsequent analyses of the effect of beer consumption on the intestinal microbiota and SCFA. According to the results regarding the amount of alcohol intake from each drink shown in Table S2, these are consistent with a moderate consumption, this being considered up to one drink (typically a can of beer, 330 mL, containing about 4% *w*/*v* alcohol) per day in women, and up to two in men [1]. It is worth pinpointing, however, that the amount of beer consumed was very similar in men and women in this study, probably because among Spanish men there is a higher prevalence of mixed drinking, including beer, wine, and spirits, and this particular pattern was excluded when selecting the current study population from the ALMICROBHOL study cohort.

Regarding the microbiota, the results showed no between-group differences in α and β diversity. The same was observed in an intervention study in which 355 mL of non-alcoholic or alcoholic beer were consumed daily by the participants. This moderate alcoholic beer consumption did not affect α or β diversity in comparison with baseline values [20]. On the other hand, in the current study, other potential confounding factors were included in the analysis of diversity, such as sex, age, or the composite BMI-fat, and no significant effect was observed for any of these in this population.

Focusing on the relative abundance of the studied taxa, a lower relative abundance of the *Clostridiaceae* family, and specifically the *Clostridium* and *Alkaliphilus* genera, was observed in individuals who consumed beer than in abstainers, while *Blautia* genus abundance was higher in beer consumers. As mentioned above, scientific evidence on the effect of beer on gut microbiota is very scarce, although there are some animal and in vitro studies on the effect of some of the main beer compounds. Thus, beer has proven to be a relatively rich source of AXOS and β-glucans. Two published works carried out in rats reported increased *Blautia* levels associated with malt, barley, or beer derived compounds [15,16]. First, in rats fed with different types of dietary fiber from barley malts, brewer’s spent grain, and barley extracts, an increase in *Blautia* was observed with the diet containing β-glucan extract [15]. This finding is coincident with the observations in the BEER group of the current study. Similarly, an increase in *Blautia* was observed in a group of high-fat diet fed rats, when barley or barley malt were incorporated to the diet, as compared to control rats on a fiber-free diet [16].

According to scientific evidence, the prebiotic intake tends to change the composition of the intestinal microbiota towards a relative increase in species belonging to *Bifidobacterium* and/or *Lactobacillus* genera. First, the endoxylanase enzymatic activity that is carried out by *Roseburia* and *Bacteroides* species degrades cereal arabinoxylans to AXOS, and these same species, together with specialized *Bifidobacterium* possessing arabinofuranosidase and xylosidase enzymes, further degrade AXOS, such as those in beer, to the monosaccharides arabinose and xylose; these leading finally to SCFA as the main fermentation output [21]. In a study based on continuous in vitro fermentation, simulating the human colon, Vardakou et al. found that the AXOS produced from the treatment of wheat with arabinoxylan endoxylanase significantly increased the levels of *Bifidobacterium* spp., while reducing the levels of *Clostridium* and *Bacteroides* [22]. In the current work, no significant changes were found in *Bifidobacterium* or *Bacteroides*, probably because the free-living human source of the samples is not directly comparable to the in vitro fermentation simulator, and the amount of AXOS would be relatively lower in real life conditions; however, a decrease in *Clostridium* was observed, in agreement with the mentioned work [21]. On the other hand, an increase in *Lactobacillus* was found with several of the malt-based ingredients or its extracts tested in rats [15]. The current results did not show differences in *Lactobacillus* levels, perhaps due to a lower amount of usable substrates in the case of moderate beer intake in the study subjects, compared to the supplemented diet in the animal model. In this sense, regarding the bifidogenic power of AXOS, supplementation studies have shown that 1.4 g AXOS for 28 days was the lowest daily dose that showed bifidogenic effects in humans [23]. Considering a moderate consumption of beer (500 mL/day) with an average amount of AXOS (range 0.49 to 1.90 g/L), it can be estimated that beer could contribute 18–68% to that minimum daily amount of 1.4 g, which might be considered a relevant contribution to the bifidogenic effect.

The *Lachnospiraceae* family is relevant among SCFA producers. While this family did not show differences between the BEER and ABS groups in the current study, several genera within the family showed significant, or almost significant, differences between the groups. The highly abundant genus *Blautia*, which has already been mentioned, and the minor genera *Pseudobutyrivibrio*, *Butyrivibrio*, and *Johnsonella*, presented higher levels in beer consumers than in abstainers. The last three genera are butyric acid producers [24,25,26]; thus, their increased abundance may be related to the increased fecal concentration of butyric acid found in the BEER group. In contrast, *Blautia* is mainly an acetate-producing bacteria [26]; however, the current study results did not show significant differences in acetate levels between groups, and no significant associations were observed between *Blautia* and acetic acid. In the only human intervention study with beer supplementation, and including microbiota and SCFA analysis, an increase in the *Bacillus* genus was found, and also in Proteobacteria such as *Pseudomonas*, *Succinivibrio*, and *Aeromonadales* [20]. Unfortunately, these observations are not in agreement with this study’s results. No differences in SCFA were found either, after the 30-day beer supplementation [20], as opposed to the butyric acid increase with moderate beer consumption reported here. The different consumption habits in individuals in the intervention study [20] and in the present observational study, involving ad-libitum consumption and a more prolonged habit, might perhaps account for the discrepancies regarding microbiota composition and SCFA production.

According to the dietary assessment results, moderate beer consumption was not associated with changes in nutrient intakes. ABS and BEER subjects showed a similar dietary profile and, specifically, the total fiber consumption, as well as the different food group contributions to fiber intake (Table S1), were similar between groups. For example, fiber from fruits and vegetables and fiber from cereals, which include abundant β-glucans and AXOS, respectively, did not differ between BEER and ABS groups, suggesting that the changes observed in the composition of the intestinal microbiota and the production of SCFA are related with the different beer intake habit.

In vitro studies performed with beer polyphenols have also been published. For example, one study suggested that (−)-epicatechin and (+)-catechin may be able to influence the gut microbiota, even in the presence of other nutrients such as carbohydrates and proteins [12]. Specifically, (+)-catechin significantly inhibited the growth of *Clostridium histolyticum,* while it promoted that of the *Clostridium coccoides-Eubacterium rectale* group, of which *Blautia coccoides* (formerly *Clostridium coccoides*) is a member. These results are in accordance with findings in the current study relative to the *Clostridium* and *Blautia* genera. In this in vitro study, *Bifidobacterium* and *Lactobacillus* spp. remained relatively unaffected, which is also in agreement with the present results [12]. Furthermore, red wine polyphenols, were also associated with significantly lower levels of *Clostridium* spp. in a murine model [10]; however higher levels of *Bacteroides*, *Bifidobacterium,* and *Lactobacillus* spp. were also reported in this study, which highlights the need to differentiate the effects of different polyphenols and alcoholic drinks, and also the exposure dose.

## 4. Materials and Methods

### 4.1. Experimental Design

This is an observational study based on a convenience sub-sample from the ALMICROBHOL study population [27]. Two hundred and sixty-one adults between 25 and 50 years, and with a BMI between 18.5 and 35 kg/m^2^, participated in the later project. The exclusion criteria in the mother study were: (1) pathological conditions such as type 1 diabetes, cancer, chronic liver, heart, kidney or lung disease, brain disorders, congenital metabolic diseases, autoimmune diseases (including thyroid disease), inflammatory bowel disease, human immunodeficiency virus (HIV), Cushing’s syndrome, or diagnosed food intolerances; (2) prescription of chronic medication; (3) antibiotics use in the last two months; (4) to be on any type of special diet; (5) having undergone a surgical procedure in the last month.

Criteria based on the alcohol consumption profiles detected in the ALMICROBHOL population were used to select the study sub-sample as follows: (1) abstainers or subjects consuming <1.5 g alcohol/day with nil beer consumption (ABS; n = 44); (2) beer consumers: ≥70% beer contribution to total alcohol consumption and 10–30 alcohol g from beer/day, while wine consumption <2 alcohol g/day and spirits <6 alcohol g/day (BEER; n = 34). These amounts of alcohol in the BEER group are equivalent to between 200 and 600 mL of beer (5% vol.) per day, while consuming less than 15 mL of wine (13% vol.) and less than 15 mL of spirits (40% vol.) per day. Thus, a total of 78 individuals were included in this study, and all of them had maintained a stable behavior regarding alcohol consumption at least for the last year.

During this observational study, subjects attended the research center twice to participate in individual interviews with trained nutritionists who collected data on their lifestyle habits. In the first visit, the nutritionist administered an ad hoc frequency recall questionnaire on alcoholic beverage consumption [28]. The questionnaire recorded the intake of wine, beer, champagne, cider, liquors, spirits, and all the mixtures by estimations over the last year. Reference drink sizes were considered. Frequency of intake was registered using a continuous scale as follows: never or almost never (0 to once every 2 months), 1 to 3 times per month, times per week, or number of times per day. Habitual intake was recorded for working days and separately for weekends. Total alcohol intake (g/d) was calculated using average grams of alcohol content per 100 mL of each alcoholic beverage. In this first visit, the overall health status, diagnosed diseases, symptoms, drug prescriptions, and sleep quality were assessed through the Spanish National Health Survey. The self-estimation of the total capital, assisted by the interviewer, was used for the socioeconomic status (SES) classification as follows: (1) “low-intermediate”: 10,000 € to 50,000 €; (2) “intermediate-high”: 50,000 € to 200,000 € and (3) “high”: above 200,000 €. Height (Soehnle), body weight, and bioimpedance analysis without shoes, and with light clothing (Tanita BC 601) were also measured in this first visit. Since the body mass index (BMI = weight (kg)/height (m^2^)) does not represent an accurate measurement of body fat, the optimal body fat percentages were considered separately for men and women; and the subjects were divided into two groups: (1) normal weight (BMI < 25 kg/m^2^) or overweight (BMI = 25–30 kg/m^2^) plus normal body fat percentages (21–32% for women; 10–20% for men); and (2) overweight (BMI = 25–30 kg/m^2^) or obese (BMI > 30 kg/m^2^) plus high body fat percentages (>32% for women; >20% for men). Cut-off criteria for body fat percentages were taken from the Tanita guidelines. In addition, subjects were instructed to collect a stool sample, in sterile conditions, and bring it frozen with the aid of cold bricks to the study center on a second visit.

During the second visit, participants completed the Minnesota Leisure-Time Physical Activity Questionnaire (MLTPAQ, Spanish version) and went through a dietary assessment. They were asked to complete a validated food frequency questionnaire, which estimates the amounts and frequency of consumption of 104 items over the past one-year period [29]. The interviewer asked for both the quantity (referred to a standard size) and the frequency of consumption of each item, which was registered as never or almost never/number of times per month (1 to 3)/number of times per week (1 to 6)/number of times a day. Consumption variability, according to the seasonal availability, especially for vegetables and fruits, was also considered. Food and beverage intake were converted into energy and nutrients using the food composition tables by Mataix et al. [30].

### 4.2. Gut Microbiota Analysis

The fecal samples from the ALMICROBHOL project were collected in sterile containers at home, immediately frozen at −20 °C and transported on the next day, in refrigerated conditions, to the study center, where they were stored at −80 °C until analyses. Starting from 180–220 mg of each fecal sample, bacterial DNA was extracted using an optimized protocol [31]. After recovery of the supernatant by centrifugation, ammonium acetate was added for protein precipitation and the resulting supernatant was treated with isopropanol during 30 min for DNA precipitation. After centrifugation, the resulting pellet was washed with 70% ethanol, dried at 37 °C until ethanol evaporation, and then washed with Tris-Ethylenediaminetetraacetic acid (EDTA) buffer. RNase was added for RNA removal, and DNA was finally recovered with the commercial QIAamp DNA Stool Mini Kit (QIAGEN GmbH), following the manufacturer’s instructions. DNA was quantified in a NanoDrop ND-1000 spectrophotometer (NanoDrop Technologies, Wilmington, DE, USA) and diluted (0.5 ng/µL) for library preparation before 16S rRNA sequencing. which involved several steps: a first polymerase chain reaction (PCR) to amplify the V3-V4 region of the 16S rRNA gene, using the primers 341F (5’-CCTACGGGNNGGCWGCAG-3’) and 785R (5’-GACTACHVGGTATCTAATCC-3’). Then, 1.5% agarose gel electrophoresis (EX 2% agarose, Invitrogen, Life Technologies, Grand Island, NY, USA) was performed to check the integrity of the amplicons and to estimate the dilution necessary for a second PCR, with adapter and barcode sequences, to facilitate sequence allocation, before loading libraries into the sequencer. After this second PCR, all the samples were run in a bioanalyzer, subsequently an equimolar pool was made, taking into account the data obtained in the bioanalyzer. This pool was cleaned with Ampure beads and run again in the bioanalyzer to check that there were no impurities. Samples sequencing was performed with a MiSeq Illumina system, using the V3 kit (Illumina, San Diego, CA, USA), and generating 2 × 270 bp reads. The analysis of microbial communities was done with the Metagenomics workflow in MiSeq Reporter (v2.3) software (San Diego, CA, USA), including demultiplexing and FASTq (text files containing sequence data with a quality score for each base) generation, obtaining 37,793,518 high-quality reads (144,803 mean reads/sample). Sequences were then clustered into operational taxonomic units (OTU) with Classify Reads, a high-performance implementation of the Ribosomal Database Project (RDP), based on the Greengenes database, obtaining 20 phyla, 243 families, 651 genera, and 1492 species (Classify Reads accuracy was 100%, 99.97%, 99.65%, and 98.65%, respectively) [32]. Taxa with relative abundance <0.001% of the total readings, and also those with a prevalence of <10 subjects, were removed, leaving a total of 20 phyla, 151 families, 364 genera, and 511 species. However, from these taxa only the beer-fermenting bacteria or their components were selected, such as the taxa belonging to *Lachnospiraceae, Ruminococcaceae, Clostridiaceae, Eubacteriaceae, Peptococacceae* families, *Bifidobacteria* spp., *Lactobacilli* spp., and *Bacteroides* genus, for statistical analysis, obtaining 3 phyla, 8 families, 36 genera, and 41 species.

Next, the raw sequences (FASTq files) related to the 78 volunteers were selected to estimate α and β diversity using mothur. From the FASTq files, the direct and indirect readings (R1 and R2) of each sample were combined. Sequences with more than 3 ambiguous bases and more than 465 bp were removed. Once this first cleaning was done, the *unique.seqs* command was executed, which groups together identical sequences and accounts for their abundance. The sequences alignment was performed with the *align.seqs* command that matches the samples’ sequences to the Greengenes reference database (May 2013 version). Non-aligned sequences were removed, and afterwards, filtering, clustering, and chimeric sequence cleaning were performed. The *classify.seqs* command was executed, which assigns the sequences a taxonomy, using the reference database; and the *remove.lineage* command, with which all those sequences that do not correspond to bacteria or archaea were eliminated, according to the taxonomic assignment. Therefore, at the end of the process, a total of 7,791,070 sequences were obtained, of which 3,222,457 were unique sequences. To start the analysis of diversity, the phylotype command was used, which clusters the sequences into phylotypes according to the taxonomic classification, and generates an OTUs abundance “.shared” file. At this point, all those OTUs that did not have more than 0.001% relative abundance were removed. Finally, the *classify.otu* command was executed, detecting a total of 189 OTUs. Rarefaction curves were generated and the α diversity indexes Chao and Shannon, were calculated using the “.shared” file. Regarding β diversity, the *dist.shared* command was executed to generate Bray-Curtis distance matrixes; as well as the *pcoa* command to generate a file that allows viewing a principal coordinates analysis (PCoA) graph in R, using the *plotPCOA* command.

### 4.3. Short Chain Fatty Acids Analysis

An aliquot of 100 mg of frozen fecal sample was diluted in 1 mL of 5% phosphoric acid, followed by homogenization and freezing of the fecal homogenates for dry matter precipitation. Stool samples were then thawed and centrifuged for 5 min at 112× *g* (Jouan Centrifuge A14, Saint Herblain, France). The SCFA acetic, propionic, butyric, isobutyric, valeric, and isovaleric acids were quantified in the supernatants by gas chromatography and flame ionization detection (GC-FID, Agilent 6890A, Agilent Technologies, Waldbronn, Germany). The capillary chromatographic column used was a DB-WAXtr column (100% polyethylene glycol, 60 m, 0.325 × 0.25) and helium was used as the carrier gas at 1.5 mL/min. Injection was made in splitless mode, with an injection volume of 1 μL and a temperature of 260 °C. Methyl valeric was used as an internal standard, and the standard curve was prepared in a similar way to the samples. The detector temperature was 260 °C. The column was heated at 50 °C for 2 min, followed by an increase of 15 °C every min to 150 °C, 5 °C every min to 200 °C, and finally 15 °C every min to 240 °C. The different SCFAs were identified by the retention time of the standard compounds.

### 4.4. Statistical Analysis

The normal distribution of the data was checked prior to analysis, and logarithmic transformation was applied for data normalization of some variables (physical activity, hours of sleep, *Clostridiaceae* family, and SCFAs). Descriptive measures used were mean ± standard deviation (SD), and median and interquartile range (IQR) for normal and non-normally distributed variables, respectively. Regarding the demographic characteristics, parametric (T test) and non-parametric (Mann–Whitney U, MW-U) tests were applied for between-group comparisons and the Chi-square test for categorical variables. General linear models, adjusted sequentially for gender, age, BMI-fat, total energy, fiber (g/1000 kcal), and MEDAS score, were used to assess the associations of beer consumption with parametric variables of the gut microbiota. Only significantly contributing factors were retained in the model. When no factor influenced the model, a one-way ANOVA test with the factor “consumption group” was used. For variables not fitting a normal distribution the MW-U test was used to compare groups defined by sex, BMI-fat status, and beer consumption, and when necessary, the population was split by sex or BMI-fat (or both) prior to beer consumption group comparison. The gut microbiota analysis was restricted to those taxa that could potentially metabolize beer compounds. Since this was a targeted analysis, no adjustment of multiple comparisons was deemed necessary. The analysis of α diversity (Chao and Shannon indexes) and SCFAs by consumption group was performed by one-way ANOVA, since other factors such as sex, age, BMI-fat, and total alcohol showed not to have an effect in the general linear model analyses. For β diversity, a Bray-Curtis distance matrix was used, and a PERMANOVA test was performed, introducing one by one, along with the “group” factor, the variables gender, age, BMI-fat, and total energy, using the adonis command from the vegan package of R. Values of *p* < 0.05 were considered significant. Data analysis was performed with SPSS software (v.23) (Chicago, IL, USA), while mothur (v.1.35.1) and R (v.3.5.3) were used to analyze α and β diversity.

## 5. Conclusions

In conclusion, this observational study on the intestinal microbiota composition in a healthy adult population revealed certain differences between regular consumers of beer in moderate amounts and non-consumers. In the absence of other dietary differences between the groups studied, moderate beer consumption was associated with higher levels of *Blautia, Pseudobutyrivibrio, Butyrivibrio,* and *Johnsonella,* and increased butyric acid, which can be attributed to the higher levels of the last three genera. In contrast, beer consumption was not associated with changes in the diversity of the gut microbiota. Intervention studies with different types of beer are needed to help provide more evidence to support the present results.

## Figures and Tables

**Table 1:** Anthropometric characteristics and lifestyle in moderate beer consumers and abstainers (by sex).

Beer Consumption Group					
**Men**	**ABS (n = 18)**	**BEER (n = 15)**	***p*^ϒ^**	*p* *	***p*^¥^**
Age (years)	37.23 (5.99)	34.05 (6.39)	0.151	--	--
BMI (kg/m^2^)	26.47 (3.20)	24.23 (2.64)	0.038	--	--
BMI-Fat (%)					
*Normal weight*	44.4	73.3	--	--	0.095
*Overweight*	55.6	26.7			
Body fat (%)	20.47 (6.32)	16.48 (5.06)	0.057	--	--
Visceral fat index	7.00 (4.00–9.00)	4.00 (3.00–6.00)	--	0.057	--
MEDAS total score	6.778 (2.264)	6.867 (1.767)	0.902	--	--
Capital (%)					
*Low (<50,000 €)*	33.3	40.0	--	--	0.848
*Medium (50,000–200,000 €)*	50.0	40.0			
*High (>200,000 €)*	16.7	20.0			
Smoking habits (%)					
*Non-smokers*	5.6	13.3	--	--	0.530
*Current smokers*	11.1	20.0			
*Former smokers*	83.3	66.7			
Physical activity (kcal/wk) ^†φ^	5588 (3793)	7280 (4497)	0.152	--	--
Sleep (h/d) ^†^	7.45 (1.05)	7.64 (0.87)	0.535	--	--
**Women**	**ABS (n = 26)**	**BEER (n = 19)**	***p*^ϒ^**	*p* *	***p*^¥^**
Age (years)	36.78 (7.18)	34.70 (6.48)	0.323	--	--
BMI (kg/m^2^)	22.80 (3.01)	23.75 (2.25)	0.253	--	--
BMI-Fat (%)					
*Normal weight*	69.2	63.2	--	--	0.670
*Overweight*	30.8	36.8			
Body fat (%)	27.60 (8.12)	29.11 (5.07)	0.480	--	--
Visceral fat index	3.00 (2.00–4.13)	4.00 (3.00–4.00)	--	0.476	--
MEDAS total score	7.269 (1.756)	7.526 (1.679)	0.624	--	--
Capital (%)					
*Low (<50,000 €)*	57.7	47.4	--	--	0.471
*Medium (50,000–200,000 €)*	30.8	47.4			
*High (>200,000 €)*	11.5	5.3			
Smoking habits (%)					
*Non-smokers*	19.2	36.8	--	--	0.339
*Current smokers*	30.8	31.6			
*Former smokers*	50.0	31.6			
Physical activity (kcal/wk) ^†φ^	3156 (2242)	2617 (2359)	0.433	--	--
Sleep (h/d) ^†^	7.75 (0.88)	7.75 (0.78)	0.943	--	--

MEDAS: Mediterranean diet adherence score. ^ϒ^ Student’s t test for parametric variables. * Mann–Whitney U (MW-U) test for non-parametric variables. ^¥^ Chi-square test for categorical variables. ^φ^ Physical activity corresponds to regular (daily/weekly/monthly) activities and excludes occasional activities. ^†^ Variables transformed to logarithmic scale (Ln). h/d, hours/day. Statistical significance was set at *p* < 0.05.

**Table 2:** Relative abundances [%] of bacterial families in moderate beer consumers and abstainers.

Beer Consumption Groups					
	ABS (n = 44)	BEER (n = 34)	*p* ^#^	*p* ^λ^	*p* *
*Lachnospiraceae*	16.16 (5.61)	17.68 (4.01)	--	0.189	--
*Ruminococcaceae*	15.16 (4.77)	15.28 (4.30)	0.958	--	--
*Clostridiaceae* ^†^	11.31 (5.93)	8.20 (3.61)	--	0.009	--
*Bacteroidaceae*	10.62 (7.89–17.71)	13.89 (7.75–20.34)	--	--	0.228
*Bifidobacteriaceae*	2.342 (0.935–5.441)	1.348 (0.516–3.260)	--	--	0.303
*Peptococcaceae*	0.268 (0.156–0.410)	0.237 (0.166–0.353)	--	--	0.323
*Eubacteriaceae*	0.146 (0.118–0.187)	0.141 (0.121–0.161)	--	--	0.465
*Lactobacillaceae*	0.134 (0.054–0.249)	0.095 (0.059–0.147)	--	--	0.181

Data shown as mean (SD) or median (interquartile range, IQR) for normally and non-normally distributed variables, respectively. ^#^ Group effect in a general linear model with fixed factors “Group” and “BMI-fat”. ^λ^ ANOVA test. * MW-U test. ^†^ Variable transformed to logarithmic scale (Ln + 1).

**Table 3:** Relative abundances [%] of bacterial genera in moderate beer consumers and abstainers.

Beer Consumption Groups			
	ABS (n = 44)	BEER (n = 34)	*p* *
***Bacteroidaceae***			
*Bacteroides*	10.62 (7.89–17.71)	13.60 (7.78–19.90)	0.268
***Lachnospiraceae***			
*Blautia*	6.419 (5.041–8.822)	8.098 (6.801–9.043)	0.044
*Lachnospira*			
*Normal weight*	2.268 (1.082–3.408)	1.951 (1.428–4.011)	0.795
*Overweight*	1.110 (0.653–1.662)	1.297 (0.992–1.822)	0.387
*Coprococcus*			
*Normal weight*	1.919 (0.957–2.580)	1.321 (0.970–1.822)	0.065
*Overweight*	1.908 (1.762–3.091)	3.223 (1.119–4.252)	0.188
*Roseburia*	1.424 (0.675–2.639)	2.196 (1.278–2.978)	0.118
*Dorea*	0.442 (0.236–0.617)	0.332 (0.233–0.570)	0.438
*Pseudobutyrivibrio*	0.224 (0.104–0.364)	0.323 (0.159–0.591)	0.037
*Butyrivibrio*	0.080 (0.034–0.179)	0.166 (0.065–0.296)	0.056
*Anaerostipes*	0.072 (0.028–0.116)	0.052 (0.007–0.121)	0.276
*Johnsonella*	0.035 (0.022–0.073)	0.055 (0.036–0.071)	0.051
*Oribacterium*			
*Normal weight*	0.019 (0.011–0.033)	0.028 (0.015–0.035)	0.200
*Overweight*	0.014 (0.009–0.023)	0.010 (0.007–0.018)	0.438
*Lachnobacterium*	0.009 (0.002–0.027)	0.014 (0.006–0.078)	0.107
*Shuttleworthia*	0.008 (0.003–0.016)	0.009 (0.002–0.017)	0.525
*Catonella*	0.001 (0.000–0.001)	0.001 (0.000–0.001)	0.674
***Ruminococcaceae***			
*Faecalibacterium*			
*Normal weight*	8.007 (4.794–9.872)	7.530 (6.330–9.767)	0.857
*Overweight*	6.067 (3.362–9.512)	6.663 (4.237–8.366)	0.842
*Ruminococcus*	4.796 (3.410–6.352)	4.019 (2.998–5.724)	0.144
*Oscillospira*	3.776 (2.436–4.839)	3.755 (2.721–4.344)	0.856
*Anaerofilum*	0.082 (0.048–0.149)	0.072 (0.049–0.131)	0.896
*Anaerotruncus*	0.059 (0.034–0.094)	0.063 (0.044–0.095)	0.643
*Ethanoligenens*	0.000 (0.000–0.001)	0.000 (0.000–0.001)	0.938
***Clostridiaceae***			
*Clostridium*	5.192 (3.514–6.378)	4.013 (3.161–5.072)	0.056
*Alkaliphilus*			
Men	2.914 (1.570–5.391)	1.535 (1.318–2.590)	0.025
Women	2.100 (0.951–3.672)	1.200 (0.463–2.608)	0.198
*Caloramator*	0.450 (0.157–1.226)	0.218 (0.080–1.144)	0.204
***Eubacteriaceae***			
*Acetobacterium*	0.144 (0.108–0.181)	0.134 (0.122–0.156)	0.724
*Eubacterium*	0.104 (0.038–0.503)	0.217 (0.017–0.981)	0.526
*Anaerofustis*	0.001 (0.000–0.003)	0.000 (0.000–0.001)	0.068
***Bifidobacteriaceae***			
*Bifidobacterium*	2.333 (0.930–5.425)	1.500 (0.509–3.804)	0.426
***Lactobacillaceae***			
*Lactobacillus*	0.131 (0.046–0.245)	0.083 (0.058–0.142)	0.201
***Peptococcaceae***			
*Desulfotomaculum*	0.070 (0.048–0.108)	0.064 (0.043–0.092)	0.450
*Peptococcus*	0.042 (0.020–0.095)	0.029 (0.018–0.073)	0.403
*Sporotomaculum*	0.024 (0.017–0.034)	0.024 (0.019–0.035)	0.896
*Desulfosporosinus*	0.023 (0.008–0.034)	0.013 (0.004–0.025)	0.090
*Dehalobacterium*	0.012 (0.001–0.028)	0.007 (0.003–0.014)	0.343
*Desulfurispora*	0.005 (0.003–0.016)	0.005 (0.003–0.008)	0.632
*Pelotomaculum*	0.003 (0.002–0.005)	0.003 (0.002–0.005)	0.545
*Desulfitobacterium*	0.001 (0.000–0.005)	0.001 (0.000–0.003)	0.402

Data shown as median (IQR). * Mann Whitney-U test. Statistical significance was set at *p* < 0.05.

**Table 4:** Relative abundances [%] of bacterial species in moderate beer consumers and abstainers.

Beer Consumption Groups			
	ABS (n = 44)	BEER (n = 34)	*p* *
*Blautia coccoides*	1.752 (1.142–2.212)	2.254 (1.724–2.912)	0.027
*Alkaliphilus peptidifermentans*	1.022 (0.270–2.407)	0.574 (0.211–1.008)	0.028
*Alkaliphilus crotonatoxidans*	0.920 (0.295–1.906)	0.376 (0.178–0.934)	0.054
*Clostridium alkalicellulosi*	0.629 (0.393–0.932)	0.668 (0.444–0.886)	0.747
*Blautia hanseii*	0.400 (0.218–0.526)	0.343 (0.285–0.492)	0.928
*Blautia wexlerae*	0.384 (0.236–0.658)	0.522 (0.341–0.819)	0.133
*Pseudobutyrivibrio xylanivorans*	0.224 (0.104–0.364)	0.323 (0.159–0.591)	0.037
*Clostridium cadaveris*	0.170 (0.072–0.577)	0.124 (0.070–0.310)	0.190
*Clostridium histolyticum*	0.144 (0.093–0.245)	0.155 (0.108–0.209)	0.904
*Clostridium frigoris*	0.102 (0.020–0.514)	0.167 (0.020–0.565)	0.657
*Butyrivibrio proteoclasticus*	0.080 (0.034–0.179)	0.166 (0.065–0.276)	0.058
*Blautia obeum*	0.079 (0.031–0.160)	0.084 (0.055–0.198)	0.328
*Clostridium caenicola*	0.056 (0.029–0.080)	0.053 (0.039–0.077)	0.832
*Clostridium hiranonis*	0.046 (0.012–0.110)	0.013 (0.002–0.038)	0.006
*Clostridium fallax*	0.038 (0.010–0.100)	0.040 (0.006–0.116)	0.687
*Johnsonella ignava*	0.034 (0.020–0.072)	0.055 (0.035–0.069)	0.046
*Clostridium thermosuccinogenes*	0.030 (0.021–0.048)	0.039 (0.020–0.116)	0.230
*Clostridium taeniosporum*	0.020 (0.013–0.049)	0.040 (0.015–0.078)	0.131
*Clostridium thermoalcaliphilum*			
Men	0.029 (0.011–0.038)	0.017 (0.011–0.044)	0.442
Women	0.016 (0.008–0.037)	0.010 (0.007–0.017)	0.103
*Clostridium termitidis*			
Men	0.011 (0.005–0.041)	0.008 (0.006–0.019)	0.442
Women	0.036 (0.013–0.066)	0.015 (0.011–0.050)	0.301
*Blautia schinkii*	0.009 (0.004–0.017)	0.010 (0.004–0.015)	0.856
*Blautia glucerasea*	0.008 (0.003–0.016)	0.010 (0.004–0.017)	0.665
*Clostridium hveragerdense*	0.008 (0.003–0.022)	0.009 (0.003–0.020)	0.956
*Clostridium cavendishii*	0.006 (0.002–0.012)	0.004 (0.003–0.008)	0.420
*Clostridium malenominatum*	0.005 (0.002–0.014)	0.004 (0.001–0.016)	0.519
*Clostridium straminisolvens*	0.003 (0.001–0.009)	0.002 (0.000–0.005)	0.263
*Clostridium proteolyticus*	0.002 (0.001–0.005)	0.002 (0.001–0.004)	0.532
*Alkaliphilus metalliredigens*	0.001 (0.000–0.002)	0.001 (0.000–0.001)	0.267
*Blautia hydrogenotrophica*	0.001 (0.000–0.006)	0.001 (0.000–0.004)	0.830
*Clostridium tepidiprofundi*	0.001 (0.000–0.002)	0.001 (0.001–0.002)	0.944
*Clostridium chartatabidum*	0.001 (0.000–0.003)	0.001 (0.000–0.002)	0.235
*Clostridium aestuarii*	0.001 (0.000–0.002)	0.000 (0.000–0.001)	0.128
*Blautia producta*	0.000 (0.000–0.003)	0.003 (0.000–0.028)	0.039

Data shown as median (IQR). * Mann Whitney-U test. Statistical significance was set at *p* < 0.05.

**Table 5:** SCFA concentration in moderate beer consumers and abstainers.

Beer Consumption Groups			
	ABS (n = 44)	BEER (n = 34)	*p* ^λ^
Acetic acid (µM/g) ^†^	33.24 (16.29)	37.79 (15.51)	0.158
Propionic acid (µM/g) ^†^	11.66 (7.08)	13.32 (6.55)	0.133
Butyric acid (µM/g) ^†^	8.831 (5.383)	11.35 (6.538)	0.032
Isobutyric acid (µM/g) ^†^	1.857 (0.942)	1.639 (0.710)	0.351
Valeric acid (µM/g) ^†^	1.854 (1.436)	1.923 (0.972)	0.376
Isovaleric acid (µM/g) ^†^	2.679 (1.691)	2.371 (1.259)	0.599

Data are shown as mean (SD). **^λ^** ANOVA test. ^†^ Variables transformed to logarithmic scale (Ln). Statistical significance was set at *p* < 0.05.

## Supplementary Materials

The following are available online. Table S1: Dietary profile in moderate beer consumers and abstainers (by sex), Table S2. Total alcohol intake and amount of alcohol intake by type of drink in moderate beer consumers and abstainers (by sex), Table S3: Relative abundances [%] of bacterial phylum in moderate beer consumers and abstainers, Figure S1: Two-dimensional scatter plot of ABS and BEER groups generated using PCoA from the Bray-Curtis distance matrix, Figure S2: Differences in *Clostridiaceae* levels between beer consumption groups, Figure S3: Differences between beer consumption groups in the levels of (A) *Alkaliphilus peptidifermentans*; (B) *Clostridium hiranonis*; (C) *Blautia coccoides*; (D) *Pseudobutyrivibrio xilanivorans*; (E) *Johnsonella ignava*; and (F) *Blautia producta*, Figure S4: Differences in the production of butyric acid between beer consumption groups.

## References

[1] de Gaetano G., Costanzo S., Di Castelnuovo A., Badimon L., Bejko D., Alkerwi A., Chiva-Blanch G., Estruch R., La Vecchia C., Panico S. (2016). Effects of moderate beer consumption on health and disease: A consensus document. Nutr. Metab. Cardiovasc. Dis..

[2] Goñi I., Díaz-Rubio M.E., Saura-Calixto F. (2008). Dietary fiber in beer: Content, composition, colonic fermentability, and contribution to the diet. Beer in Health and Disease Prevention.

[3] Pérez-Jiménez J., Díaz-Rubio M.E., Saura-Calixto F. (2013). Non-extractable polyphenols, a major dietary antioxidant: Occurrence, metabolic fate and health effects. Nutr. Res. Rev..

[4] Neish A.S. (2009). Microbes in gastrointestinal health and disease. Gastroenterology.

[5] Hansen N.W., Sams A. (2018). The microbiotic highway to health—New perspective on food structure, gut microbiota, and host inflammation. Nutrients.

[6] Engen P.A., Green S.J., Voigt R.M., Forsyth C.B., Keshavarzian A. (2015). The gastrointestinal microbiome: Alcohol effects on the composition of intestinal microbiota. Alcohol Res. Curr. Rev..

[7] Bach Knudsen K.E. (2015). Microbial degradation of whole-grain complex carbohydrates and impact on short-chain fatty acids and health. Adv. Nutr..

[8] Maslowski K.M., Vieira A.T., Ng A., Kranich J., Sierro F., Yu D., Schilter H.C., Rolph M.S., MacKay F., Artis D. (2009). Regulation of inflammatory responses by gut microbiota and chemoattractant receptor GPR43. Nature.

[9] Tolhurst G., Heffron H., Lam Y.S., Parker H.E., Habib A.M., Diakogiannaki E., Cameron J., Grosse J., Reimann F., Gribble F.M. (2012). Short-chain fatty acids stimulate glucagon-like peptide-1 secretion via the G-protein-coupled receptor FFAR2. Diabetes.

[10] Dolara P., Luceri C., De Filippo C., Femia A.P., Giovannelli L., Caderni G., Cecchini C., Silvi S., Orpianesi C., Cresci A. (2005). Red wine polyphenols influence carcinogenesis, intestinal microflora, oxidative damage and gene expression profiles of colonic mucosa in F344 rats. Mutat. Res. Fundam. Mol. Mech. Mutagen..

[11] Quesada-Molina M., Muñoz-Garach A., Tinahones F.J., Moreno-Indias I. (2019). A new perspective on the health benefits of moderate beer consumption: Involvement of the gut microbiota. Metabolites.

[12] Tzounis X., Vulevic J., Kuhnle G.G.C., George T., Leonczak J., Gibson G.R., Kwik-Uribe C., Spencer J.P.E. (2008). Flavanol monomer-induced changes to the human faecal microflora. Br. J. Nutr..

[13] Possemiers S., Bolca S., Grootaert C., Heyerick A., Decroos K., Dhooge W., De Keukeleire D., Rabot S., Verstraete W., Van de Wiele T. (2006). The Prenylflavonoid Isoxanthohumol from Hops (*Humulus lupulus* L.) Is Activated into the Potent Phytoestrogen 8-Prenylnaringenin In Vitro and in the Human Intestine. J. Nutr..

[14] Paraiso I.L., Plagmann L.S., Yang L., Zielke R., Gombart A.F., Maier C.S., Sikora A.E., Blakemore P.R., Stevens J.F. (2019). Reductive Metabolism of Xanthohumol and 8-Prenylnaringenin by the Intestinal Bacterium *Eubacterium ramulus*. Mol. Nutr. Food Res..

[15] Teixeira C., Prykhodko O., Alminger M., Fåk Hållenius F., Nyman M. (2018). Barley Products of Different Fiber Composition Selectively Change Microbiota Composition in Rats. Mol. Nutr. Food Res..

[16] Zhong Y., Nyman M., Fåk F. (2015). Modulation of gut microbiota in rats fed high-fat diets by processing whole-grain barley to barley malt. Mol. Nutr. Food Res..

[17] Cummings J.H. (1997). Short-chain fatty acid enemas in the treatment of distal ulcerative colitis. Eur. J. Gastroenterol. Hepatol..

[18] Rivière A., Selak M., Lantin D., Leroy F., De Vuyst L. (2016). Bifidobacteria and Butyrate-Producing Colon Bacteria: Importance and Strategies for Their Stimulation in the Human Gut. Front. Microbiol..

[19] Ramirez-Farias C., Slezak K., Fuller Z., Duncan A., Holtrop G., Louis P. (2009). Effect of inulin on the human gut microbiota: Stimulation of *Bifidobacterium adolescentis* and *Faecalibacterium prausnitzii*. Br. J. Nutr..

[20] Hernández-Quiroz F., Nirmalkar K., Villalobos-Flores L.E., Murugesan S., Cruz-Narváez Y., Rico-Arzate E., Hoyo-Vadillo C., Chavez-Carbajal A., Pizano-Zárate M.L., García-Mena J. (2020). Influence of moderate beer consumption on human gut microbiota and its impact on fasting glucose and β-cell function. Alcohol.

[21] Broekaert W.F., Courtin C.M., Verbeke K., van de Wiele T., Verstraete W., Delcour J.A. (2011). Prebiotic and other health-related effects of cereal-derived arabinoxylans, arabinoxylan-oligosaccharides, and xylooligosaccharides. Crit. Rev. Food Sci. Nutr..

[22] Vardakou M., Nueno Palop C., Gasson M., Narbad A., Christakopoulos P. (2007). In vitro three-stage continuous fermentation of wheat arabinoxylan fractions and induction of hydrolase activity by the gut microflora. Int. J. Biol. Macromol..

[23] Na M.H., Kim W.K. (2007). Effects of Xylooligosaccharide Intake on Fecal Bifidobacteria, Lactic acid and Lipid Metabolism in Korean Young Women. Korean J. Nutr..

[24] Moore L.V.H., Moore W.E.C. (1994). *Oribaculum catoniae* gen. nov., sp. nov.; *Catonella morbi* gen. nov., sp. nov.; *Hallella seregens* gen. nov., sp. nov.; *Johnsonella ignava* gen. nov., sp. nov.; and *Dialister pneumosintes* gen. nov., comb. nov., nom. rev., anaerobic gram-negative bacilli from the human gingival crevice. Int. J. Syst. Bacteriol..

[25] Kopečný J., Zorec M., Mrázek J., Kobayashi Y., Marinšek-Logar R. (2003). *Butyrivibrio hungatei* sp. nov. and *Pseudobutyrivibrio xylanivorans* sp. nov., butyrate-producing bacteria from the rumen. Int. J. Syst. Evol. Microbiol..

[26] Rajilić-Stojanović M., de Vos W.M. (2014). The first 1000 cultured species of the human gastrointestinal microbiota. FEMS Microbiol. Rev..

[27] Redondo-Useros N., Gheorghe A., Díaz-Prieto L.E., Villavisencio B., Marcos A., Nova E. (2019). Associations of Probiotic Fermented Milk (PFM) and Yogurt Consumption with *Bifidobacterium* and *Lactobacillus* Components of the Gut Microbiota in Healthy Adults. Nutrients.

[28] Nova E., San Mauro-Martín I., Díaz-Prieto L.E., Marcos A. (2019). Wine and beer within a moderate alcohol intake is associated with higher levels of HDL-c and adiponectin. Nutr. Res..

[29] Martin-Moreno J.M., Boyle P., Gorgojo L., Maisonneuve P., Fernandez-Rodriguez J.C., Salvini S., Willett W.C. (1993). Development and validation of a food frequency questionnaire in Spain. Int. J. Epidemiol..

[30] Mataix J. Tablas de Composición de Alimentos [Food Composition Tables]. http://www.sennutricion.org/es/2013/05/11/tablas-de-composicin-de-alimentos-mataix-et-al.

[31] Dore J., Ehrlich S.D., Levenez F., Pelletier E., Alberti A., Bertrand L., Bork P., Costea P.I., Sunagawa S., Guarner F. (2015). IHMS_SOP 06 V1: Standard Operating Procedure for Faecal Samples DNA Extraction, Protocol, Q. International Human Microbiome Standards.

[32] 16S Metagenomics App Illumina. https://support.illumina.com/help/BaseSpace_App_16S_Metagenomics_help/16S_Metagenomics_App_Help.htm.

