# Supplementary material for: Association of Moderate Beer Consumption with the Gut Microbiota and SCFA of Healthy Adults
Source: Molecules. 2020 Oct 17;25(20):4772. doi: 10.3390/molecules25204772 (PMC7587552; doi:10.3390/molecules25204772)
Supplement: Supplementary file 1 [file molecules-25-04772-s001.pdf]

Table S1. Dietary profile in moderate beer consumers and abstainers (by sex).

Beer consumption groups				
**Men**	**ABS (n=18)**	**BEER (n=15)**	**P^{Υ}**	**P^{*}**
Energy (kcal/d)	2243 (836)	2092 (537)	0.550	--
Carbohydrates (%)	42.29 (5.19)	40.28 (8.11)	0.415	--
Fiber (g/1000kcal)	11.1 (4.0)	10.0 (3.3)	0.409	--
Fiber (g/d)	24.3 (9.7)	21.2 (10.2)	0.383	--
Fiber from vegetables (g/d)	5.3 (3.5-8.0)	5.5 (3.6-7.9)	--	0.957
Fiber from fruit (g/d)	5.1 (2.7-9.6)	2.5 (1.6-5.0)	--	0.145
Fiber from cereals (g/d)	4.3 (2.7-7.0)	3.4 (2.8-6.0)	--	0.709
Fiber from legums (g/d)	3.3 (2.0-3.8)	2.6 (1.1-3.7)	--	0.486
Fiber from other (olive, nuts, etc.) (g/d)	3.1 (2.2-5.1)	1.7 (0.9-3.4)	--	0.145
Fiber from beer (g/d)	0.0 (0.0-0.0)	0.5 (0.4-0.7)	--	**<0.001**
Proteins (%)	17.75 (2.15)	17.20 (2.85)	0.533	--
Fat (%)	39.63 (5.22)	38.40 (6.26)	0.543	--
Monounsaturated fat (%)	17.31 (3.51)	17.64 (4.49)	0.814	--
Polyunsaturated fat (%)†	6.16 (1.29)	5.62 (1.81)	0.227	--
Saturated fat (%)	11.10 (3.04)	10.92 (1.43)	0.836	--
Alcohol (%)	0.26 (0.00-0.38)	4.19 (2.89-5.48)	--	**<0.001**
**Women**	**ABS (n=26)**	**BEER (n=19)**	**P^{Υ}**	**P^{*}**
Energy (kcal/d)	1901 (447)	2186 (516)	*0.054*	--
Carbohydrates (%)	39.76 (8.15)	38.14 (4.63)	0.402	--
Fiber (g/1000kcal)	11.9 (3.5)	10.60 (2.07)	0.144	--
Fiber (g/d)	22.1 (7.7)	22.9 (6.1)	0.723	--
Fiber from vegetables (g/d)	7.1 (5.3-10.1)	8.8 (5.9-9.7)	--	0.581
Fiber from fruit (g/d)	3.7 (2.4-5.3)	4.0 (1.6-5.0)	--	0.982
Fiber from cereals (g/d)	3.7 (2.2-4.7)	4.0 (2.1-5.1)	--	0.573
Fiber from legums (g/d)	2.1 (1.7-3.3)	2.8 (1.5-3.9)	--	0.613
Fiber from other (olive, nuts, etc.) (g/d)	2.6 (1.8-4.3)	2.3 (1.7-4.0)	--	0.730
Fiber from beer (g/d)	0.0 (0.0-0.0)	0.6 (0.5-0.8)	--	**<0.001**
Proteins (%)	16.93 (3.06)	16.34 (2.40)	0.483	--
Fat (%)	42.97 (7.05)	40.95 (4.30)	0.277	--
Monounsaturated fat (%)	19.55 (4.59)	19.01 (3.46)	0.667	--
Polyunsaturated fat (%)†	6.21 (1.62)	5.68 (1.49)	0.290	--
Saturated fat (%)	11.94 (2.94)	11.59 (2.16)	0.663	--
Alcohol (%)	0.0 (0.0-0.66)	3.92 (2.92-5.19)	--	**<0.001**

ΥStudent's t test for parametric variables. *Mann-Whitney U test for non-parametric variables. †Variables transformed to logarithmic scale (Ln). Statistical significance was set at P<0.05. φTotal dietary fiber is represented as the amount of fiber from beer (estimated [2]) plus dietary fiber obtained in the dietary analysis.

Deleted: CER

Deleted: CER

Table S2. Total alcohol intake and amount of alcohol intake by type of drink in moderate beer consumers and abstainers (by sex).

	Beer consumption groups		
**Men**	**ABS (n=18)**	**BEER (n=15)**	**P***
Total alcohol (g/d)	0.38 (0.0-0.73)	14.67 (12.57-24.28)	**<0.001**
Beer (alcohol g/d)	0.0 (0.0-0.0)	11.57 (10.29-17.13)	**<0.001**
Wine (alcohol g/d)	0.0 (0.0-0.49)	0.0 (0.0-1.57)	0.259
Spirits (alcohol g/d)	0.0 (0.0-0.09)	1.07 (0.54-2.86)	**<0.001**
**Women**	**ABS (n=26)**	**BEER (n=19)**	**P***
Total alcohol (g/d)	0.0 (0.0-0.71)	14.67 (12.39-21.47)	**<0.001**
Beer (alcohol g/d)	0.0 (0.0-0.0)	13.5 (11.14-19.18)	**<0.001**
Wine (alcohol g/d)	0.0 (0.0-0.10)	0.39 (0.0-0.79)	**0.018**
Spirits (alcohol g/d)	0.0 (0.0-0.09)	0.36 (0.0-0.71)	**0.014**

Data are shown as Median (IQR). *Mann-Whitney U test. Statistical significance was set at P<0.05

Table S3. Relative abundances [%] of microbial phylum in moderate beer consumers and abstainers.

	Beer consumption groups			
	**ABS (n=44)**	**BEER (n=34)**	**P#**	**P***
Firmicutes	60.27 (9.49)	59.98 (9.07)	0.805	--
Bacteroidetes	22.57 (8.72)	24.74 (9.95)	0.221	--
Actinobacteria	3.20 (1.34-6.23)	2.14 (0.98-4.25)	--	0.359

#"Group" effect in a general linear model. The Firmicutes model was adjusted for gender and the Bacteroidetes model was adjusted for total energy (kcal/day). *Mann-Whitney U test for non-parametric variables. Statistical significance was set at P<0.05.

Deleted: 2

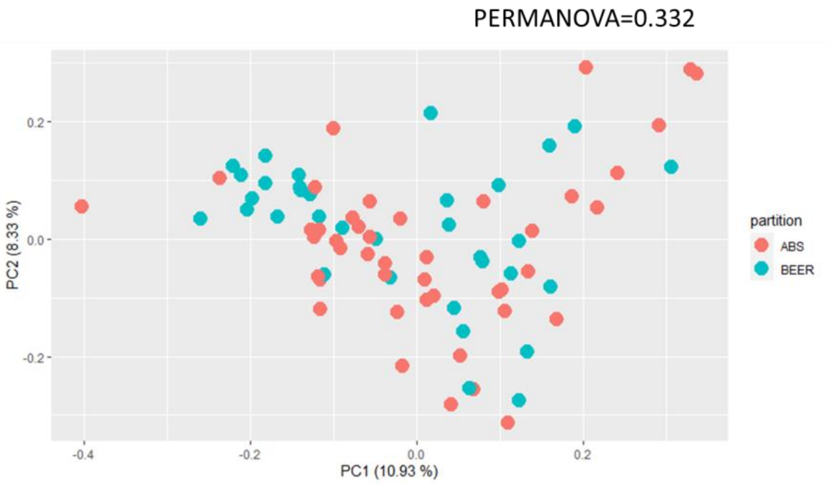

Figure S1. Two-dimensional scatter plot of ABS and BEER groups generated using PCoA from the Bray-Curtis distance matrix. The PERMANOVA test was performed, introducing one by one, along with the “group” factor, the variables gender, age, BMI-fat and total energy, using the adonis command from the vegan package of *R*.

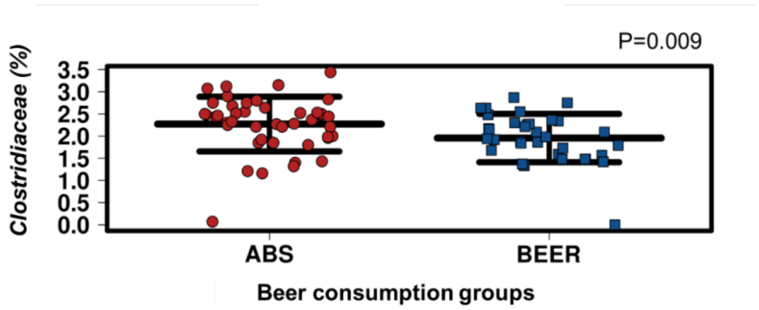

Figure S2. Differences in *Clostridiaceae* levels between beer consumption groups. ANOVA test. P <0.05.

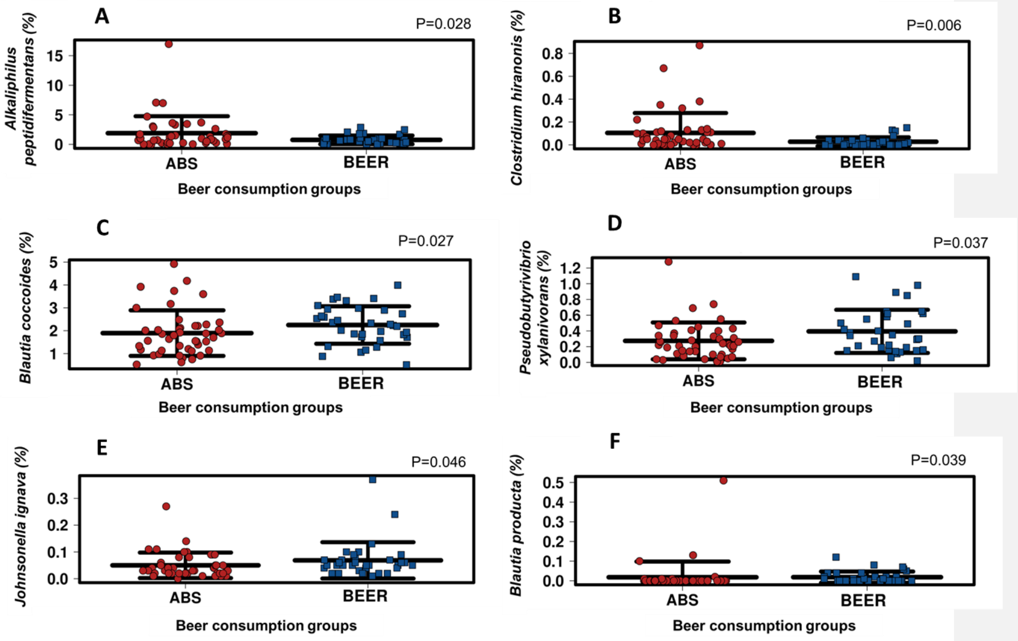

Figure S3. Differences between beer consumption groups in the levels of A) *Alkaliphilus peptidifermentans*; B) *Clostridium hiranonis*; C) *Blautia coccoides*; D) *Pseudobutyrivibrio xilanivorans*; E) *Johnsonella ignava*; and F) *Blautia producta*. MW-U test. P <0.05.

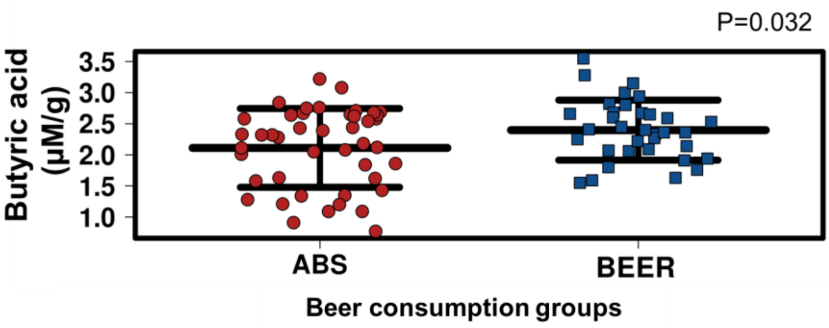

Figure S4. Differences in the production of butyric acid between beer consumption groups. ANOVA test. P <0.05.
